# Comparison of Local Recurrence Among Early Breast Cancer Patients Treated With Electron Intraoperative Radiotherapy vs Hypofractionated Photon Radiotherapy an Observational Study

**DOI:** 10.3389/fonc.2018.00207

**Published:** 2018-06-05

**Authors:** Marina Guenzi, Elisabetta Bonzano, Renzo Corvò, Francesca Merolla, Alice Pastorino, Francesca Cavagnetto, Stefania Garelli, Carlo Alberto Cutolo, Daniele Friedman, Liliana Belgioia

**Affiliations:** ^1^Department of Radiation Oncology, IRCCS Policlinico San Martino, Genoa, Genoa, Italy; ^2^Health Science Department (DISSAL), University of Genoa, Genoa, Genoa, Italy; ^3^Department of Medical Physics, IRCCS Policlinico San Martino, Genoa, Genoa, Italy; ^4^DiNOGMI, University of Genoa, Genoa, Genoa, Italy; ^5^Department of Surgery, IRCCS Policlinico San Martino and University, Genoa, Genoa, Italy

**Keywords:** early breast cancer, intraoperative radiotherapy, external beam radiotherapy, whole-breast irradiation, accelerated partial breast irradiation

## Abstract

**Purpose:**

To evaluate local recurrence (LR) in women with early breast cancer (BC) who underwent intraoperative radiation therapy with electrons particles (IORT-E) or adjuvant hypofractionated external radiotherapy (HYPOFX).

**Materials and methods:**

We retrospectively analyzed 470 patients with early BC treated at our center from September 2009 to December 2012. 235 women were treated with breast-conserving surgery and immediate IORT-E (21 Gy/1 fraction) while 235 patients underwent wide excision followed by hypofractionated whole-breast irradiation. Radiotherapy modality was chosen according to an individualized decision based on tumor features, stage, technical feasibility, age, and acceptance to be enrolled in the IORT-E group.

**Results:**

After a median follow-up of 6 years, we observed 8 (3.4%) and 1 (0.42%) LR in the IORT-E and in the HYPOFX group (*p* = 0.02), respectively. The two groups differed in the prevalence of clinical characteristics (*p* < 0.05): age, tumor size, surgical margins, receptors, ki67, and histology. 4 and 1 woman in the IORT-E and HYPOFX group died of BC, respectively (*p* = 0.167). OS and DFS hazard ratio [HR] were 2.14 (95% IC, 1.10–4.15) and 2.09 (95% IC, 1.17–3.73), respectively.

**Conclusion:**

Our comparison showed that IORT-E and HYPOFX are two effective radiotherapy modalities after conservative surgery in early BC. However, at 6 years a significant higher rate of LR occurred in patients submitted to IORT-E with respect to HYPOFX. This finding may be correlated to some subsets of patients who, depending on the biological characteristics of the BC, may be less suitable to IORT-E.

## Introduction

Breast cancer (BC) is the most common cancer in women. The increasing use of mammographic screening enables an early diagnosis to be made in most cases. Conservative surgery is the procedure of choice in the management of early-stage BC, and all guidelines indicate whole-breast radiotherapy as a part of conservative treatment.

The administration of 50 Gy in 25 fractions over 5 weeks to the whole breast was considered the standard until a few years ago, when the publication of the long-term results of important British and Canadian randomized studies proved the effectiveness and efficiency of schemes administered over shorter times (hypofractionated radiotherapy) (1, 2).

Furthermore, in the past 10 years, the possibility of treating only the tumor bed has been analyzed, as ipsilateral breast tumor recurrences (IBTR) developed in and around the tumor bed in 44–86% of cases (3), and treatment of the rest of the breast might be unnecessary. Indeed, by limiting irradiation to the area of potential recurrence by means of partial breast irradiation (PBI), much of the surrounding tissues (including the lung, heart, uninvolved ipsilateral breast) could be spared, thereby reducing toxicity and improving the cosmetic outcome (4, 5).

Several techniques of PBI exist: brachytherapy (intracavitary and interstitial approaches), external beam radiation using either three-dimensional conformal radiotherapy or intensity modulated radiation therapy, and proton radiation. However, all these procedures involve the use of several radiation fractions, requiring the patients to attend the Radiotherapy Department on several days.

This difficulty can be overcome by another PBI modality, namely intraoperative radiation therapy with an electron beam (IORT-E), which selectively treats the breast volume in which the tumor was located by delivering a single dose of radiation at the time of surgery, after complete removal of the tumor. This procedure reduces treatment time and, potentially, improves the patient’s quality of life.

Here, we present a retrospective comparison between two treatment modalities used in our center: the single administration of 21 Gy by means of IORT-E, and whole-breast irradiation (WBI) according to a hypofractionated external radiotherapy (HYPOFX) (39 Gy delivered in 13 fractions over 3 weeks to the whole breast, plus an individualized concomitant boost to the tumor bed up to 42–43 Gy) (6). The comparison was made on 470 consecutive women with early BC, who were treated from 2009 to 2012 with either IORT-E or HYPOFX. This comparison was prompted by our observations over a median follow-up of more than 6 years. The women analyzed in this research displayed the same patient and tumor characteristics before surgery. Our aims were to evaluate the clinical outcomes of these two modalities in a large series of patients, and to better understand how to select women for the best adjuvant radiotherapy.

## Materials and Methods

Patients were selected for either IORT-E or HYPOFX according to a multidisciplinary discussion involving mainly radio-oncologists and breast surgeons. The radiotherapy modality was chosen according to an individualized decision based on the clinical features of the tumor, stage, breast size, technical feasibility of IORT-E, age, and patients’ willingness to be enrolled in the IORT-E group.

Patients were informed that IORT-E was an investigational modality (Clinical Trial. Gov NCT01276938) approved by Ethics Committee of IRCCS Policlinico San Martino Hospital of Genoa (approval number OR09.001), while hypofractionated radiotherapy was the common practice in breast adjuvant therapy in our institution.

### IORT-E Modality

We analyzed the treatments and their results in 235 consecutive patients who underwent wide excision (with sentinel node biopsy) followed by IORT with electron particles (IORT-E) to the tumor bed as the sole radiation modality between September 2009 and December 2012. Patients signed an informed consent form specifying the pros and cons of IORT-E.

#### Patient Selection

At the time of patient selection (2009) there were no ASTRO and ESTRO selection criteria yet. The candidates for IORT-E were identified in accordance with the criteria of the American Brachytherapy Society and American Society of Breast Surgeons (>50 years), T less than 2 cm, pN0, infiltrating ductal carcinoma or ductal carcinoma *in situ*, and microscopically negative margins (>2 mm) (7), regardless of the biological features of the tumor. However, publication of the ESTRO and ASTRO criteria prompted us to implement more restrictive patient selection (8, 9). The characteristics of patients treated with IORT-E are summarized in Table 1.

**Table 1:** Characteristics of patients in intraoperative radiation therapy with an electron beam (IORT-E) group and HYPOFX group.

	IORT-E, *n*(%)	HYPOFX *n*(%)	*p* Value
Number	235	235	

**Age, years**			0.0007
<50 50–59 60–69 ≥70	8 (3.4%) 27 (11.5%) 57 (24.3%) 143 (60.9%)	45 (19.1%) 103 (43.8%) 87 (37%)	

**Lesion size, mm**			0.0103
≤10 10–15 15–20 >20	56 (23.8%) 87 (37%) 74 (31.5%) 18 (7.7%)	110 (46.8%) 48 (20.4%) 46 (19.6%) 31 (13.2%)	

**Surgical margins, status**			0.0001
Negative Positive Close	228 (97%) 3 (1.3%) 4 (1.7%)	207 (88.1%) 1 (0.4%) 27 (11.5%)	

**Nodal status**			0.4427
Negative Positive Missing	227 (96.6%) 7 (3.0%) 1 (0.4%)	221 (94.0%) 10 (4.3%) 4 (1.7%)	

**Estrogen receptor**			0.5490
Absent Present Missing	7 (3.0%) 226 (96.2%) 2 (0.9%)	5 (2.1%) 230 (97.9%) 0	

**Progesterone receptor**			<0.0001
Absent Present Missing	165 (70.2%) 67 (28.5%) 3 (1.3%)	33 (14.0%) 202 (86.0%) 0	

**HER2**			0.1815
Over-expressed (+++) Not over-expressed (0, +, ++) Missing	10 (4.3%) 222 (94.5%) 3 (1.3%)	5 (2.1%) 230 (97.9%) 0	

**Proliferation index (Ki-67), %**			<0.0001
<14 14–19 ≥20 Missing	68 (28.9%) 60 (25.5%) 105 (44.7%) 2 (0.9%)	127 (54.0%) 55 (23.4%) 52 (22.1%) 1 (0.4%)	

**T**			0.05
1 2	217 (92.3%) 18 (7.7%)	204 (86.8%) 31 (13.2%)	

**Grading**			0.6581
1 2 3	39 (16.6%) 176 (74.9%) 20 (8.5%)	42 (17.9%) 178 (75.7%) 15 (6.4%)	

**Histology**			0.0196
Invasive ductal carcinoma Invasive lobular carcinoma Other	191 (81.3%) 19 (8.1%) 25 (10.6%)	166 (70.6%) 35 (14.9%) 34 (14.5%)	

#### Surgery

The patients treated underwent wide excision with negative margins (5 mm) and negative sentinel node biopsy (no axillary dissection was performed). Most patients underwent wide excision with negative margins; in seven patients margins were found close or positive, due to discrepancies between initial intraoperative pathology evaluation on the frozen section, and the results on the definitive examination on the fixed section. In nine patients, lymph node micro-invasion was detected in the definitive histological examination performed after surgery. After lumpectomy, the wide mobilization of the mammary gland, from the fascia of the pectoralis major and from the skin allows preparation of the clinical target volume to be treated.

To spare the underlying healthy tissue (muscle, rib, lung, and heart), a dedicated disk of lead and aluminum is inserted into the space between the gland and the pectoral muscle.

A sterile applicator is introduced through the skin incision and placed directly in contact with the breast target. To prevent herniation, a film is applied to the breast tissue at the far end of the applicator (10).

#### Radiotherapy Technique

A single dose of 21 Gy, prescribed at 90% of the isodose was delivered by a dedicated linear accelerator (LIAC–SIT Sordina IORT technology S.p.A., Italy). This accelerator is equipped with perspex cylindrical applicators of different diameters (from 4 to 10 cm). An applicator of an appropriate diameter is selected according to the size of the tumor, the length of the incision, and the size of the breast. The median applicator diameter was 6 cm (range, 4–7 cm). Mobile LIAC deliver electrons at different nominal energies: 4–6–8–10 MeV. The appropriate beam energy is chosen on the basis of gland thickness, as estimated in at least three different points. Moreover, to avoid superficial under-dosage due to dose build-up at the beam entrance (−5.4%: 4 MeV; −5.6%: 6 MeV; −3.1%: 8 MeV; −0.7%: 10 MeV), we prefer to use nominal beam energies higher than 8 MeV. Thus, therapeutic ranges for 5 cm applicator diameter are: 4 MeV: 12 mm, 6 MeV: 15 mm, 8 MeV: 20 mm, and 10 MeV: 26 mm.

Intraoperative radiation therapy with an electron beam minimizes the dose to the normal surrounding tissues, owing to the dose deposition characteristics of the electron beam, and thoracic wall irradiation can be limited by inserting a steel-polytetrafluoroethylene (PTFE) (3 mm + 3 mm) shielding plate between the deep side of the residual breast gland and the pectoral muscle; the plate diameter must be at least 1 cm (preferably 2 cm) greater than the diameter of the applicator (10).

#### Dosimetry and Quality Assurance of IORT-E Delivery

Linear accelerator Linac was calibrated by means of a Fricke dosimeter in inter-comparison with two plane-parallel ion chambers (11). MicroMOSFET 502-RDM (Best Medical Canada Ltd.) detectors were employed to monitor the exit dose, defined as the dose at the deepest part of the target: detectors are placed inside a thin, sterile catheter (6Fr closed-end brachytherapy catheter), and fixed to the center of the PTFE side of the shielding disk before insertion into the breast. To perform treatment, optimized monitor units were compared by means of the method described by Agostinelli et al. (12): irradiation is split into two phases in order to allow an appropriate action level so as to re-check the relative position between the applicator and shielding disk or to correct the dose if the first metal-oxide semiconductor field-effect transistor (MOSFET) reading is higher than 10%. In this case, the second phase of irradiation is performed by using self-normalization in order to cover 95% of the volume with 95% of the prescribed dose suggested by Agostinelli et al. (12).

### Hypofractionated External Radiotherapy (HYPOFX)

We analyzed the treatment and results of 235 consecutive patients with the same clinical characteristics as those of the IORT-E group. The HYPOFX group underwent wide excision followed by hypofractionated WBI, which was planned and delivered during the same period (2009–2012) (6). Table 1 summarizes the characteristics of patients treated with HYPOFX.

HYPOFX patients received 39 Gy, which was delivered to the whole breast in 3-Gy fractions over 3 weeks; they also received a concomitant boost dose of 3–4 Gy, which was delivered in 3–4 fractions to the lumpectomy cavity once a week. Four fractions per week is the schedule commonly used in our Department in order to optimize clinical activities and Linac quality control. On the basis of the Linear-Quadratic model, we assumed that 39 Gy in 3 Gy fractions was equivalent to 50 Gy in 2.0 Gy fractions. This radiotherapy schedule had previously been tested in clinics, and preliminary results were published in 2010 (6). The procedures of treatment planning have been described previously (6).

In the planning phase, the patient was positioned on a wing-board and a computerized tomography (CT GE Lightspeed Ultra) scan was performed. Four tattoos were created in order to properly position the patient during each treatment session.

Whole-breast irradiation was performed by means of 3D conformal radiation therapy with tangential beams and sub-fields to reduce hotspots (preferably not more than 105% of the whole-breast prescription dose). Irradiation of the boost volume was carried out with two or three fields, not necessarily with the same tangential-field isocenter. Organs at risk (OARs) were shielded by using an MLC collimator. For plan optimization, we considered OARs dose constraints for the ipsilateral lung, heart, and left anterior descending coronary artery.

We optimized boost planning in plan sum (whole-breast plan plus boost plan) in order to reduce the margins of boost fields.

## Comparison Endpoints

The primary endpoint of this comparative matched analysis was to evaluate local recurrence (LR) rates at 6 years in pts who had undergone intraoperative radiation therapy and in pts treated with hypofractionated external radiotherapy. Secondary end-points were overall survival (OS) and disease-free survival (DFS) rates for the two different radiotherapy modalities.

## Statistical Methods

The cumulative incidence of LR and OS plots and DFS were drawn by means of the Kaplan–Meier analysis method. The log-rank test was used to assess the difference between patients treated with intraoperative radiation therapy and patients treated with hypofractionated external radiotherapy. Multivariable Cox proportional hazard regression was used to adjust the hazard ratios of LR between the groups for demographic and disease characteristics. Statistical analysis was performed by means of MedCalc 17.9.7 (MedCalc Software, Ostend, Belgium).

## Results

**Figure 1:**
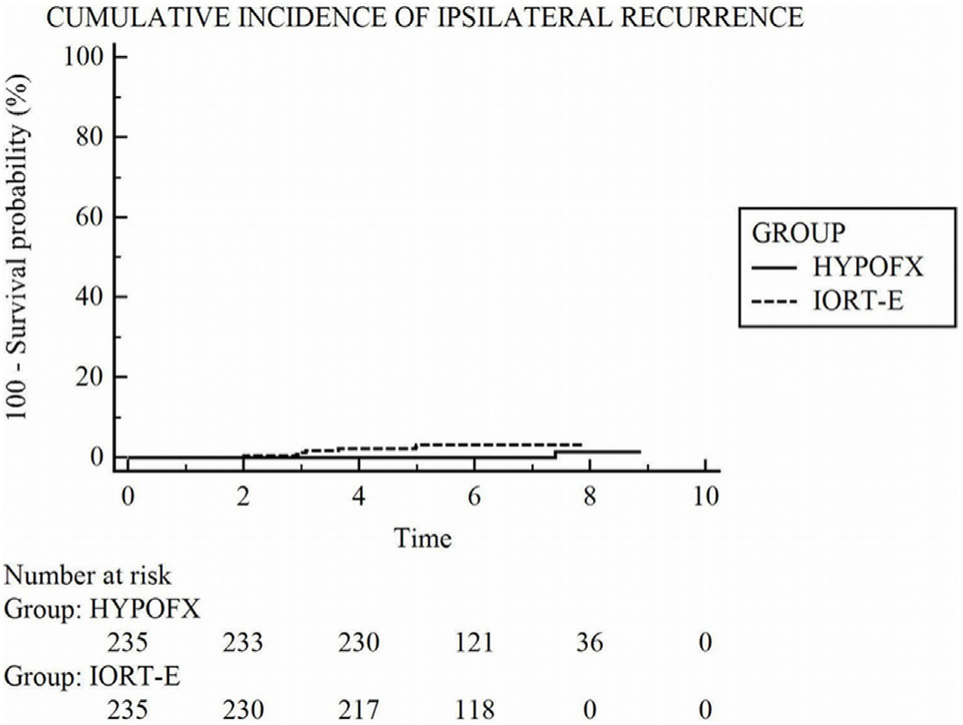
Kaplan–Meier estimates of the cumulative probability of ipsilateral recurrence.

After a median follow-up of 6.1 (5.4–7.0) years in the IORT-E group and 6.0 (5.4–7.7) years in the HYPOFX group, we observed local relapses in 3.4% (8/235) of IORT-E patients and 0.42% (1/235) of HYPOFX patients (*p* = 0.0192) (Figure 1). All eight patients who developed IBTR during follow-up had adverse biological risk factors: high histological grade, ki67 value >20, invasive lobular carcinoma (Table 2). No patient with a luminal A tumor suffered relapsed. Only one patient relapsed in the HYPOFX group (Table 2). A total of 23 IORT-E pts and 12 HYPOFX pts died. Of the IORT-E patients who died, 4 (17%) died of BC and 19 (83%) of other causes: three of heart disease, six of a second tumor, three of non-cancer-related causes, and seven of factors related to age (60% were aged >70 years). Of the 12 HYPOFX patients who died, only one (8%) died of BC and 11 (92%) of other causes: one of brain ischemia, one of cholangiocarcinoma, one of pancreatic cancer, one of Parkinson’s disease, two of heart failure, and five of age-related factors (Table 3).

**Table 2:** Characteristics of patients who developed ipsilateral breast tumor recurrences (IBRT) in intraoperative radiation therapy with an electron beam (IORT-E) group and in HYPOFX group.

	Histology	T (mm)	Grade	ER%	PgR%	KI67%	HER2
IORT-E	LCI	12	G2	92	4	2	0
	LCI	16	G2	96	95	28	0
	DCI	18	G3	99	84	83	0
	DCI	25	G3	12	0	27	3+
	DCI	15	G2	97	6	24	0
	DCI	15	G2	99	83	36	0
	DCI	13	G3	99	45	82	1+
	DCI	25	G3	92	85	34	1+

HYPOFX	MUCINOUS	9	G2	97	97	26	1+

**Table 3:** Mortality rate in intraoperative radiation therapy with an electron beam (IORT-E) group and in HYPOFX group.

	IORT-E (*n* = 235)	HYPOFX (*n* = 235)	*p* Value
Died of breast cancer	4 (1.7)	1 (0.4)	0.167
Died of other causes	19 (8.1)	11 (4.7)	0.133
Overall death	23 (9.8)	12 (5.1)	

*Data are numbers (%). Chi-square test*.

**Figure 2:**
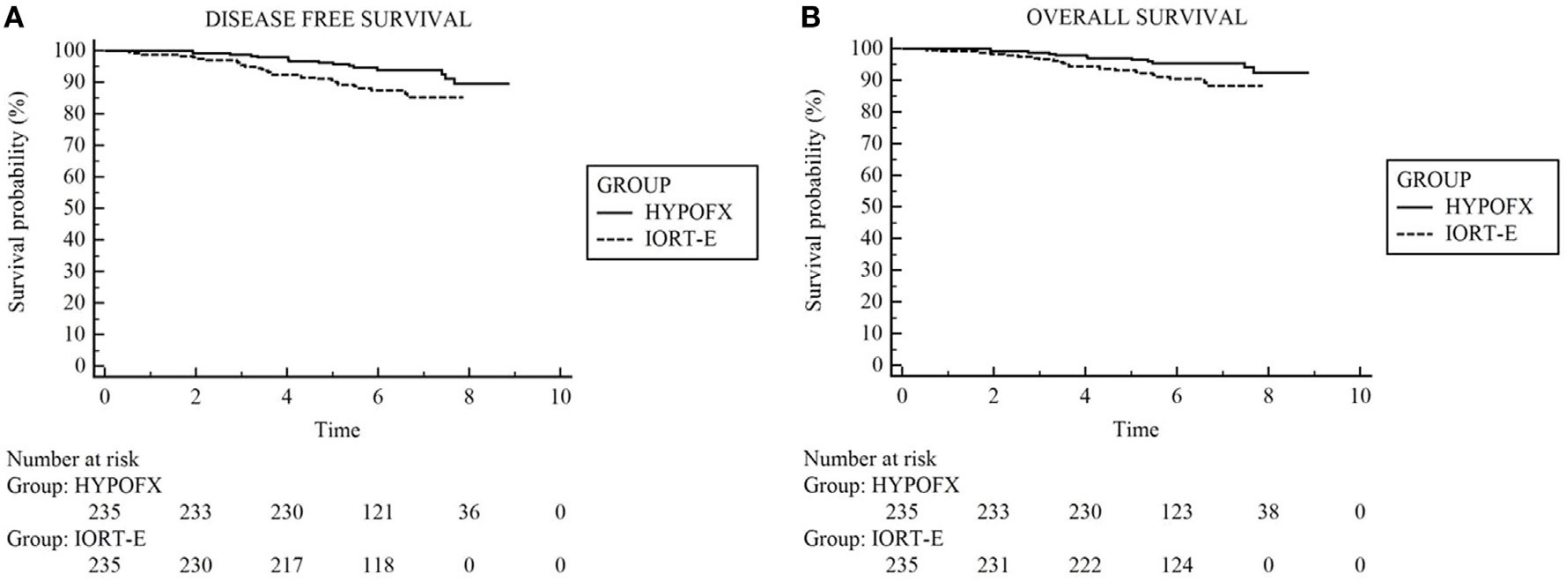
The disease-free survival and overall survival (OS) using Kaplan–Meier method. **(A)** Disease-free survival, **(B)** OS.

As shown by the Chi-Square test, the difference between the mortality rates in the two groups was not significant. We used the log-rank test to analyze OS and DFS in the two groups: differences in both OS and DFS between the two groups were significant only on unadjusted analysis (Table 4). Figures 2A,B shows DFS and OS.

**Table 4:** Unadjusted and adjusted hazard ratios (HRs) in patients treated with intraoperative radiation therapy with an electron beam (IORT-E) compared with HYPOFX.

	Unadjusted		Adjusted	
	HR	*p* Value	HR	*p* Value
Ipsilateral breast tumor recurrences recurrence	8.0231 (2.0000–32.1844)	0.0192	6.2216 (0.6432–60.1769)	0.1144
Overall survival	2.1387 (1.1014–4.1528)	0.0274	0.9320 (0.4255–2.0414)	0.8603
Disease-free survival	2.0934 (1.1734–3.7346)	0.0138	1.0436 (0.5256–2.0644)	0.9024

## Discussion

In recent years, various strategies have been introduced in order to optimize patients’ quality of life and healthcare system resources. PBI and the use of hypofractionated schemes are among the most studied and applied options.

In comparison with other PBI techniques, IORT offers the advantage of being delivered during surgery, with the possibility to directly visualize the target, thereby maximizing the precision of dose delivery to the target volume and sparing surrounding healthy tissue (breast, lung, heart, and skin).

Administering radiation treatment during surgery improves the quality of life of patients, as they do not have to attend the radiotherapy center to receive many fractions of external beam radiation (10). Furthermore, from a radiobiological point of view, the radiation dose is delivered before tumor cells have a chance to proliferate, when the tissues are richly vascularized, which potentially makes them more sensitive to the action of the radiation (oxygen effect) (13).

Our analysis compared IORT-E treatment with hypofractionated radiation therapy administered in the same period to consecutive patients with early BC. The outcomes may be interesting as, to our knowledge, no similar data have been published. One randomized controlled trial compared IORT-E with a standard radiotherapy schedule (50 Gy in 25 fractions). However, although hypofractionated radiotherapy is considered the standard of care in early BC in most centers, no phase III trial has compared PBI with this shortened fractionated regimen. Thus, the widespread adoption of hypofractionated radiation therapy, which is strongly supported by literature data (ASTRO), should prompt radio-oncologists to compare hypofractionated radiation therapy with PBI such as IORT-E.

In our analysis of 470 patients, we detected a statistically significant difference in IBTR between the IORT-E and HYPOFX groups (3.4 vs 0.4%; *p* = 0.0192). We are aware that patients in HYPOFX group presented higher biological risk factors (high histological grade, ki67 value >20, invasive lobular carcinoma—Table 2), despite this IBRT was lower than in IORT-E group. This could be partly explained by the fact that these patients underwent to WBI with an additional higher boost dose of 4 Gy, concomitantly delivered to the lumpectomy cavity, with a consequent high dose delivered to the whole breast that might have reduce the LR rate.

Moreover this is a retrospective non-randomized comparison in patients treated in the same period of time and assigned to undergo IORT-E HYPOFX on the basis of technical feasibility or patient preference, despite all these limits the outcomes may provide preliminary suggestions for the choice of the best radiotherapy technique (IORT-E vs HYPOFX). Interestingly, we observed that all eight patients who developed IBTR in the IORT-E group had adverse biological risk factors: high histological grade, ki67 value >20, and invasive lobular carcinoma, while no patients with a luminal A tumor suffered relapse. Not all the IORT-E patients analyzed underwent tru-cut biopsy before surgery, since the procedure has only recently become a standard of care and, as reported by several authors, an aggressive biological subtype is a strong predictor of disease relapse (14–16). For this reason, the IORT-E group included a large number of high-grade tumors and not luminal A. These aggressive characteristics are significantly correlated with the likelihood to develop LR; on this basis we strongly underline that a preliminary tru-cut biopsy is deeply indicated.

Only after the publication of the ASTRO and ESTRO consensus statements the eligibility criteria for PBI selection become more accurate. Moreover, some patients were assigned to the IORT-E group on account of their poor general condition or the presence of diseases, including psychiatric conditions that did not allow any other treatment. These issues may explain the higher rate of IBTR in the IORT-E group.

Researchers from The European Oncology Institute (IEO), Milan, Italy, who are leading experts in IORT-E, have described their retrospective experiences in several papers (14–18), in which they analyzed the various procedures and discussed their results in the light of the criteria set out in the ASTRO and ESTRO consensus statements (19, 20).

Analysis of their data reveals that the 5-year rate of ipsilateral BC recurrence is certainly higher in the “unsuitable” and “cautionary” ASTRO categories than in the “suitable” category (8.8; 4.4; and 1.5%, respectively) (*p* = 0.0003) (19).

The results of the ELIOT randomized trial, published in 2013 by Veronesi et al., show a higher rate of IBTR in the intraoperative radiotherapy group than in the external standard radiotherapy group: the 5-year event rate for IBRT was 4.4% (95% CI 2·7–6·1) in the former and 0.4% (0·0–1·0) in the latter (21). These data seem to be comparable to ours. Even if it is not a randomized study, the results obtained in our analysis, are consistent with the European Institute of Oncology randomized data (Veronesi et al.) (21).

Silverstein discussed the results of the ELIOT study on classifying the patients into low- and high-risk groups on the basis of tumor size, receptor status, nodal positivity, and grade. Low-risk women (69.4% of ELIOT patients) had a 5-year IBTR rate of only 1.5%, as opposed to 11.3% in the 30.6% of ELIOT patients with 1 or more high-risk factors.

As confirmed by previous findings (19), proper selection for IORT-E is mandatory (22).

With regards to EBRT, in our department we have progressively introduced several hypofractionated schemes, considering the results of British and Canadian trials. Analysis of 10-year results of the UK START trials confirms that an appropriate hypofractionated radiotherapy schedule is safe and effective in women with early BC; furthermore, normal tissue effects, such as breast shrinkage, telangiectasia, and breast edema, were significantly less common in the hypofractionated group than in the standard group. These results suggest that the administration of 40 Gy delivered in 15 fractions should be continued, and a hypofractionated 3-week schedule has already been adopted by most UK centers as the standard adjuvant radiotherapy (2).

The data from the START trials are in agreement with those from the Canadian study.

The Ontario trial showed that local tumor control and cosmetic breast cancer results were no worse with a hypofractionated scheme (42.5 Gy in 16 fractions over 3.2 weeks) than with standard treatment (50 Gy in 25 fractions over 5 weeks). The risk of LR at 10 years was 6.7% in the standard-treatment patients and 6.2% in the hypofractionated regimen. Both in the control group and in the hypofractionated group, the majority of patients achieved a good or excellent esthetic outcome (71.3 and 69.8%) (23). Moreover, hypofractionated RT can improve patients’ quality of life by reducing access to the center, to accelerate patient turnover, and to save healthcare resources.

In our Hypofx WBI group, we recorded a 0.42% local relapse rate (1/235 pts). This datum is extremely interesting, as it confirms literature data and demonstrates that our schedule is similarly effective and safe. The improved techniques enabled by new advanced linear accelerators, and better OAR contouring, allow toxicity to be reduced, particularly the high level of cardiotoxicity in pts who undergo hypofractionated radiotherapy treatment on the left side (24).

## Conclusion

Our analysis demonstrated that the IORT-E technique is efficacious; the most important finding is that, in some subsets of patients, it may be equivalent than other techniques. Indeed, patient selection must be rigorous, in that the benefit yielded by this treatment depends on the biological characteristics of the tumor. In those patients who are suitable for IORT-E (i.e., high estrogen receptor, low ki67 index, and low grade), the technique can be suggested and the pros and cons of the single-dose therapy discussed with the patient, not least with regards to the better quality of life offered by this procedure.

Moreover, this investigation confirms that the adjuvant hypofractionated scheme is a favorable and well-tolerated strategy that provides excellent local control. In pts who present unfavorable biological characteristics on tru-cut, this approach may be better.

Only a randomized comparison between IORT-E and HYPOFX could confirm our suggestions and estimate the level of evidence and the strength of recommendation of IORT vs hypofractionated regimens in clinical practice.

## Ethics Statement

This study was exempt from ethical approval procedures because it is a retrospective data analyses.

## Author Contributions

MG and EB: contributed equally to this work, wrote the paper, and reviewed final manuscript. FM and AP: data collection. FC and SG: review and editing of final manuscript. Provided insight into how results relate to medical physic. CC: data analysis, ran all statistical tests. RC and LB: review and editing of final manuscript. Provided insight into how results relate to radiation oncology. DF: review and editing of final manuscript. Provided insight into how results relate to surgical oncology.

## Conflict of Interest Statement

The authors declare that the research was conducted in the absence of any commercial or financial relationships that could be construed as a potential conflict of interest.

## Abbreviations

IBTR, ipsilateral breast tumor recurrences; PBI, partial breast irradiation; 3D-CRT, three-dimensional conformal radiotherapy; IMRT, intensity modulated radiation therapy; IORT-E, intraoperative radiation therapy with an electron beam; WBI, whole-breast irradiation; HYPOFX, hypofractionated external radiotherapy; CTV, clinical target volume; LIAC, mobile linear accelerators; PTFE, polytetrafluoroethylene; MOSFET, metal-oxide-semiconductor field-effect transistor; OARs, organs at risk; LADCA, left anterior descending coronary artery; LR, local recurrence; DFS, disease-free survival; OS, overall survival.
